# High Diversity of *vacA* and *cagA Helicobacter pylori* Genotypes in Patients with and without Gastric Cancer

**DOI:** 10.1371/journal.pone.0003849

**Published:** 2008-12-03

**Authors:** Yolanda López-Vidal, Sergio Ponce-de-León, Gonzalo Castillo-Rojas, Rafael Barreto-Zúñiga, Aldo Torre-Delgadillo

**Affiliations:** 1 Programa de Inmunología Molecular Microbiana, Departamento de Microbiología y Parasitología, Facultad de Medicina, Universidad Nacional Autónoma de México (UNAM), Mexico City, Mexico; 2 Unidad de Epidemiología Clínica, Instituto Nacional de Ciencias Médicas y Nutrición Salvador Zubirán (INCMNSZ), Mexico City, Mexico; 3 Departamentos de Endoscopia y Gastroenterología. Instituto Nacional de Ciencias Médicas y Nutrición Salvador Zubirán (INCMNSZ), Mexico City, Mexico; The Research Institute for Children at Children's Hospital New Orleans, United States of America

## Abstract

**Background:**

*Helicobacter pylori* is associated with chronic gastritis, peptic ulcers, and gastric cancer. The aim of this study was to assess the topographical distribution of *H. pylori* in the stomach as well as the *vacA* and *cagA* genotypes in patients with and without gastric cancer.

**Methodology/Principal Findings:**

Three gastric biopsies, from predetermined regions, were evaluated in 16 patients with gastric cancer and 14 patients with dyspeptic symptoms. From cancer patients, additional biopsy specimens were obtained from tumor centers and margins; among these samples, the presence of *H. pylori vacA* and *cagA* genotypes was evaluated. Positive *H. pylori* was 38% and 26% in biopsies obtained from the gastric cancer and non-cancer groups, respectively (*p* = 0.008), and 36% in tumor sites. In cancer patients, we found a preferential distribution of *H. pylori* in the fundus and corpus, whereas, in the non-cancer group, the distribution was uniform (*p* = 0.003). A majority of the biopsies were simultaneously *cagA* gene-positive and -negative. The fundus and corpus demonstrated a higher positivity rate for the *cagA* gene in the non-cancer group (*p* = 0.036). A mixture of *cagA* gene sizes was also significantly more frequent in this group (*p* = 0.003). Ninety-two percent of all the subjects showed more than one *vacA* gene genotype; s1b and m1 *vacA* genotypes were predominantly found in the gastric cancer group. The highest *vacA*-genotype signal-sequence diversity was found in the corpus and 5 cm from tumor margins.

**Conclusion/Significance:**

High *H. pylori* colonization diversity, along with the *cagA* gene, was found predominantly in the fundus and corpus of patients with gastric cancer. The genotype diversity observed across systematic whole-organ and tumor sampling was remarkable. We find that there is insufficient evidence to support the association of one isolate with a specific disease, due to the multistrain nature of *H. pylori* infection shown in this work.

## Introduction

*Helicobacter pylori* is a gastric pathogen that infects nearly 50% of the world's population [1]. It is considered the major etiologic agent of chronic active gastritis and is generally accepted as the primary cause of peptic ulcer disease; it is also a known risk factor for gastric cancer [2]. Gastric cancer development has been demonstrated to span several decades, beginning sequentially with *H. pylori* infection and the development of chronic active gastritis [3]. Glandular atrophy and intestinal metaplasia eventually occur in a subset of patients and terminate in gastric cancer [4]. *H. pylori* infection has been suggested to lead to an increased relative risk (RR)—on the order of four to nine-fold—for developing precancerous gastric conditions, especially when the infection occurs during childhood [5]. Given that only a minority of *H. pylori*-infected individuals develop this disease, it is thought that the development of disease is dependent on the expression of specific bacterial virulence genes, for example *cagA* and *vacA* [6], both of which are highly expressed regardless of the disease state of the host [7], [8]. The associations between gastric topography, the genotypic diversity of the *H. pylori vacA* and *cagA* genes, and the development of gastric cancer remain controversial. The aim of the present research was to evaluate the distribution of the *vacA* and *cagA* genotypes of *H. pylori* along the stomach in patients with and without gastric cancer.

Hypothesis: a higher frequency of *H. pylori* is associated with early stages of gastric cancer.

## Methods

Patients referred for endoscopic study due to suspicion of cancer or dyspeptic symptoms were invited to participate in a cross-sectional prospective study. All patients with cancer were included, as well as those with non-ulcer dyspepsia and/or gastro-esophageal reflux. Exclusion criteria included coagulation abnormalities, non-steroid anti-inflammatory (NSAID) drug intake within the last 72 h, portal hypertension, and the presence of lymphoma in the histological study.

All patients signed a written informed consent form prior to participation in the study. The study protocol was approved by the Institutional Review Board of the Instituto Nacional de Ciencias Médicas y Nutrición Salvador Zubirán (INCMNSZ), registry number CIBH-1081.

Upper gastrointestinal endoscopy was performed according to a standard technique. A systematic biopsy-sampling scheme was used as follows: from each patient, three gastric biopsies were obtained from each of the four stomach sites (antrum, angular portion, corpus, and fundus). From the patients with gastric cancer, additional biopsy specimens were obtained from the center and margin of the tumor and at a distance of at least 2–5 cm from the tumor margins.

All pathologic samples from patients with gastric cancer were evaluated by a single experienced pathologist and classified according to the Lauren classification as diffuse, intestinal, or mixed type [9]. The description of advanced gastric cancer was based on the Borrmann classification [10]. Morphological early gastric cancer sub-types were classified according to Japanese Endoscopy Society guidelines [11], [12].

*H. pylori* was cultured by smearing biopsy specimens on the surface of horse blood agar plates (10% horse blood in Casman agar base [BBL Microbiology Systems, Cockeysville, MD, USA]) [8].

Chromosomal DNA was extracted [13], and the *H. pylori 16S rRNA* and *cagA* gene was PCR amplified as previously described [7]. The *vacA* signal sequence and mid-region were typed by allelic type-specific PCR, as described previously by Morales *et al.* [8].

*cagA* variable region gene amplification. The *cagA* 3′ repeat regions of the *H. pylori* strains studied in this investigation were classified according to their length (due to the presence of a variable number of repeat sequences in the 3′ region of the gene). The amplification products obtained were categorized by size into one of three groups: short (906 bp); medium (1,008 bp), and long (1,110 bp), according to Karita *et al*. [14]. Amplification was carried out using primers cagRV17-F 5′-acc ctg gtc ggt aat gga tta tct-3′ and cag22-R 5′-tta aga ttt ttg gaa acc acc ttt t-3′, which were designed based on sequences retrieved from Genbank using ALIG (scientific and educational software) and ClustalW (BCM Search Launcher, multiple sequence alignments).

Data were analyzed for descriptive purposes utilizing relative frequencies and arithmetic means (standard deviation [SD]), as deemed appropriate. Comparisons among groups were performed by χ^2^ analysis, as well as with Fisher-Freeman-Halton exact test for contingency tables. The gastric cancer group was compared to patients with non-ulcer dyspepsia and/or gastroesophageal reflux. Cancer patients were stratified as early for TNM stage 0, I, or II and advanced for III or IV. The alpha level was set at 0.05. Given that multiple simultaneous comparisons were conducted, a nominal alpha-value correction was introduced following the Bonferroni procedure. Consequently, we regarded associations with *p* values between 0.05 and 0.01 as suspect. In contrast, results at the *p* ≤0.0063 level were considered reasonably significant. Values between 0.01 and 0.0063 were regarded as marginal. All analyses were performed using Stata statistical software (Intercooled Stata 7.0 for Windows 98/95/NT Package, 2002) and Stat-Xact (v. 4.0.1., Cytel Software Corp., 1999).

## Results

Thirty-two patients were included in this study. Based on endoscopic diagnosis, 18 were classified into the gastric cancer group and 14 into the non-cancer group. Due to histological evidence of gastric lymphoma, however, two patients in the gastric cancer group had to be excluded, leaving 16 people in that group. The average age of the patients in the gastric cancer and dyspeptic groups was 57.6±16.7 years (56% male) and 47.2±13.3 years (14% male), respectively. Ten patients (63%) displayed a diffuse tumor according to the Lauren classification. Five patients had early gastric cancer (type I, three; II, one; IIB, one) and 11 had advanced gastric cancer (type IIIA, four; IIIB, three; IV, four), according to the Japanese Research Society for Gastric Cancer guidelines and the Borrmann classification.

In our 411 biopsy samples, *H. pylori* was identified by either culture or PCR in 38% (78/203) of the biopsy samples from the gastric cancer group, 26% (54/208) of the non-cancer group (*p* = 0.008), and 36% (40/110) of the tumor sites (see Tables 1 and 2). *H. pylori* demonstrated a differential topographic distribution in patients with gastric cancer, with a higher positivity rate in the fundus and corpus (*p* = 0.003), table 1. The non-cancer group showed no differential topographic distribution. A somewhat higher positivity of *H. pylori* was found in the tumor sites by culture or at tumor margins by PCR; however, these increases in positivity were not statistically significant (Table 2). There was no significant correlation between the *H. pylori* positivity rate and TNM cancer stage (early vs. late).

**Table 1:** *H. pylori* positivity distribution in biopsies from patients with or without gastric cancer

Anatomic site	Culture		*P* value	PCR-*16S rRNA*		*P* Value	Culture+PCR-*16S rRNA*		*P* value
	Non-cancer +^a^/biopsies (%)	Gastric cancer +^a^/biopsies (%)		Non-cancer +^a^/biopsies^b^ (%)	Gastric cancer +^a^/biopsies^b^ (%)		Non-cancer +^a^/biopsies (%)	Gastric cancer +^a^/biopsies (%)	
Fundus	12/42 (29)	24/42 (57)		1/10 (10)	1/6 (17)		13/52 (25)	25/48 (52)	0.007
Corpus	11/42 (26)	21/42 (50)		3/10 (30)	3/6 (50)		14/52 (27)	24/48 (50)	0.023
Angular portion	12/42 (29)	15/45 (33)		1/10 (10)	3/10 (30)		13/52 (25)	18/55 (33)	NS
Antrum	12/42 (29)	9/42 (21)		2/10 (20)	2/10 (20)		14/52 (27)	11/52 (21)	NS
Total	47/166 (28)	69/171 (40)	0.022	7/40 (18)	2/10 (20)	NS	54/208 (26)	78/203 (38)	0.008
*P* value	NS	0.003		NS	NS		NS	0.003	

a Positivity for *Helicobacter pylori*;

b pool of three gastric biopsies from each anatomic site that were culture-negative. NS = not significant.

**Table 2:** *H. pylori* positivity distribution in biopsies from tumor sites of patients with early or advanced gastric cancer

Anatomic site	Culture +^a^/biopsies (%)	PCR-*16S rRNA* +^a^/biopsies^b^ (%)	Culture+PCR-*16S rRNA* +^a^/biopsies (%)
Mid-tumor	5/16 (31)	5/12 (42)	10/28 (36)
Tumor margin	6/16 (38)	6/11 (55)	12/27 (44)
At least 2 cm**	6/16 (38)	3/11 (27)	9/27 (33)
At least 5 cm**	5/16 (31)	4/12 (33)	9/28 (32)
Total	22/64 (34)	18/46 (39)	40/110 (36)
*P* value	NS	NS	NS
*P* value (trend)	NS	NS	NS
*P* value (early vs. advanced)	NS	NS	NS

a Positivity for *Helicobacter pylori*;

b gastric biopsies that were culture-negative.

** At least 2 and 5 cm away from the tumor margin. NS = not significant.

We found *H. pylori-cagA* positivity in 72% of the gastric samples from the gastric cancer group, 73% of the non-cancer group (*p* = NS) (Table 3), and 83% of the tumor sites (*p* = not significant [NS]). The *H. pylori-cagA* topographic distribution was not statistically significant in the gastric cancer-group, while the non-cancer group demonstrated a higher positivity rate in the fundus and corpus; however, the significance of this finding is questionable (*p* = 0.036) (Table 3). Again, in the tumor sites, we found no significant distribution pattern for the *cagA* positivity fraction, nor was there a difference between patients with early and advanced gastric cancer.

**Table 3:** *H. pylori cagA*-positive distribution in biopsies from patients with or without gastric cancer

Anatomic site	*H. pylori cagA* a		*P* value
	Non-cancer +^a^/biopsies (%)	Gastric cancer +^a^/biopsies (%)	
Fundus	12/13 (92)	17/25 (68)	NS
Corpus	14/16 (88)	17/26 (65)	NS
Angular portion	8/13 (62)	14/18 (78)	NS
Antrum	7/14 (50)	11/13 (85)	NS
Total	41/56 (73)	59/82 (72)	NS
*P* value	0.036	NS	

a Only strains and biopsies samples that were positive (primary culture) for *H. pylori* based on 16S ribosomal RNA PCR were considered. NS = not significant.

Eighty-one *H. pylori* strains were studied (80 *cagA* +), including 46 strains from patients with gastric cancer and 35 from non-cancer patients. Only two *cagA* gene sizes were obtained from the 80 *H. pylori* strains studied; none of the longer *cagA* sequences were found. In both groups, the shorter *cagA* was the most frequently detected. The results are summarized in Table 4. Interestingly, both *cagA* sizes were amplified from 12 (15%) strains, suggesting that these patients were co-infected with multiple *H. pylori* strains. This finding was obtained more often in the non-cancer patient group (28.6%) than in the gastric cancer group (4.4%) (*p* = 0.003).

**Table 4:** *cagA* size variation in *H. pylori* strains isolated from gastric cancer and non-cancer patient groups*

PCR fragment		Non-cancer group *n* = 35 (%)	Gastric cancer group *n* = 46 (%)
906 bp	1,008 bp		
Absence	Absence	0	1 (2.17)
Absence	Presence	9 (25.71)	8 (17.39)
Presence	Absence	16 (45.71)	35 (76.09)
Presence	Presence	10 (28.57)	2 (4.35)

* Cancer vs Non-cancer groups Fisher's exact test P = 0.003.

**Figure 1:**
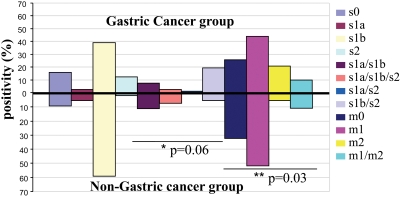
Signal sequence and middle region allele of the *vacA* gene obtained from patients with and without gastric cancer.

The predominant signal sequence genotype in patients with gastric cancer, present in 39% of the group, was s1b. The s1b/s2 combination was present in 20% of the group and s2 was present in 12%. Sixteen percent of patients with gastric cancer had an unidentifiable signal sequence (s0). There was no statistically significant distribution of *vacA* genotypes across the stomach (*p* = NS) (see Figure 1). The most frequent *vacA* genotype in the non-cancer group was s1b, which was present in 59% of the group, followed by the s1a/s1b combination in 11%, s1a/s1b/s2 in 7%, and an unidentifiable signal sequence in 9% (*p* = 0.06), Figure 1. Analysis of the topographic genotype distribution showed that genotype s1b of *vacA* was predominant in all zones (fundus, corpus, angular portion, and antrum) of the stomach in both groups studied (Figure S1). We found a distinct and marginally significant difference in the distribution patterns of these genotypes between the cancer and non-cancer groups (*p* = 0.01). Both the corpus and fundus of the gastric cancer group exhibited a greater diversity of signal-sequence genotypes, as did the corpus in the non-cancer group (Figure S1).

**Figure 2:**
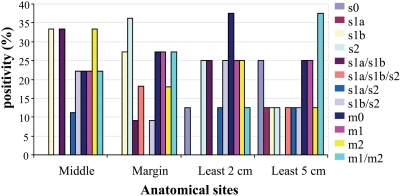
Signal sequence and middle region allele distribution of the *vacA* gene in the tumor site.

**Figure 3:**
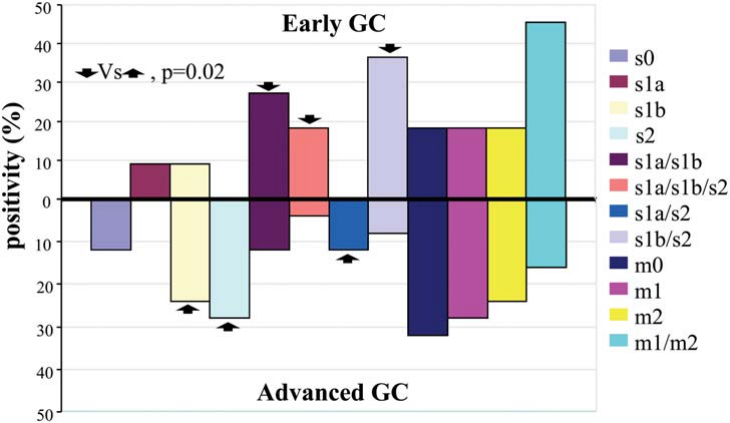
Signal sequence and middle region allele distribution of the *vacA* gene in the tumor site of patients with early and advanced gastric cancer.

The most frequent *vacA* genotypes for each of the tumor sites (center and margin of the tumor, and at least 2–5 cm from the tumor margins) were s1b and s2, which were present in 19%; s1a/s1b and s1b/s2 were each present in 17%, and s1a/s1b/2, s1a/s2, and s0 were present in 8%. There was no significant association between the genotype and tumor site proximity (Figure 2). The distribution of this genotype in tumor and anatomic sites in patients with cancer was not statistically significant (*p* = 0.06) (Figures 1 and 2). A significant association (*p* = 0.02) between genotype and tumor stage was identified: s1a/s1b, s1a/s1b/s2, and s1b/s2 genotypes were predominant in the early gastric cancer group, whereas s1b, s2, and s1a/s2 genotypes were relatively abundant in the advanced gastric cancer group (Figure 3).

The most frequent mid-region genotype in patients with gastric cancer was m1, which was present in 44% of the patients, followed by m0 (untypeable genotype) in 26%, m2 in 21%, and the m1/m2 combination in 10% (Figure 1). In the non-cancer group, the most frequent *vacA* genotype was m1, which was present in 52% of the patients, followed by m0 in 32%, the m1/m2 combination in 11%, and m2 in 5%; there was a distinct distribution pattern for these genotypes, with a questionable significance level (*p* = 0.03) (Figure 1). Analysis of the topographic distribution across anatomic zones of the mid-region genotype showed that the m1 genotype was predominantly (50%) in the angular portion, with the remaining in the fundus (48%) and the corpus (42%). The m0 genotype was predominantly in the antrum (39%) in the gastric cancer group (Figure S1). In the non-cancer group, the m1 genotype was most abundant in the fundus (77%); 56% was in the corpus, and 43% was in the antrum. The m0 genotype was detected in the angular portion (62%), with no statistically significant difference between study groups (Figure S1). A nearly uniform distribution of the different genotypes was found in the tumor sites, (Figure 2). There was no difference between anatomic zones and tumor sites in patients with gastric cancer (Figure 1 and 2) or between early and advanced tumors (Figure 3).

**Figure 4:**
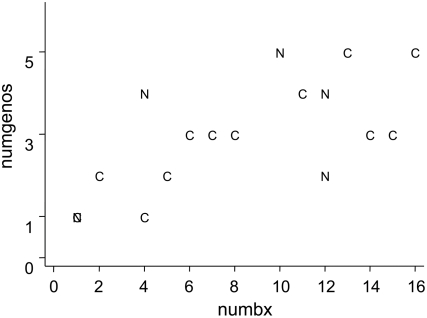
Distinct genotype number (numgeno) of the *vacA* gene signal segment according to the total number of biopsies (numbx) obtained from subjects exhibiting the presence of *H. pylori* present and with and without cancer (C = cancer group; N = non-cancer group).

Table 5 shows the analysis of the distinct *vacA* genotypes found within each individual for whom more than one biopsy was studied. Ninety-two percent (12/13) of the cancer patients had more than one genotype present. A similar finding was observed in the non-cancer group. Three subjects in the non-cancer group were consistently positive for the *cagA* gene, and another three possessed both types (positive and negative) simultaneously. In the cancer group, six individuals were positive for *cagA* and seven for both types. No subjects were consistently negative for this gene. Figure 4 displays the number of biopsies per individual and the number of distinct *vacA* gene signal segment (SS) genotypes. The lowest number of genotypes in patients with 10 or more biopsies was two, and the highest was five.

**Table 5:** Variability in the *vacA* genotypes (SS and MR regions) per subject across the entire biopsy sampling scheme for the stomach in patients who were positive for *H. pylori* and had at least two analyzable biopsies (maximum = 16 gastric cancer patients and 12 non-cancer subjects)

	*vacA* genotypes			
	Signal sequence		Mid-region	
	Number of distinct genotypes per subject	Number of subjects	Number of distinct genotypes per subject	Number of subjects
Gastric cancer group	1	1	1	1
	2	3	2	6
	3	4	3	5
	4	1	4	1
	5	4	-	-
	6-8	0	-	-
	TOTAL	13	TOTAL	13
Non-cancer group	1	0	1	1
	2	2	2	3
	3	0	3	0
	4	2	4	2
	5	1	-	-
	6–8	0	-	-
	TOTAL	5	TOTAL	6

## Discussion

The present study resulted in four major findings: first, *H. pylori* is uniformly distributed across the stomach in patients having only dyspeptic symptoms; second, *H. pylori* has a preference for the fundus and the corpus in patients with gastric cancer; third, in patients with dyspeptic symptoms, *H. pylori-cagA* has a preferential distribution in the fundus and corpus and there is a high frequency of mixed *cagA* sizes; fourth, the s1b genotype was predominant in both study groups, and the s1a genotype was present only in the dyspeptic group.

The high *H. pylori* positivity in the fundus of patients with gastric cancer may have been due to its proximity to the corpus, a zone that is colonized in *H. pylori*-induced gastritis. Corpus-predominant atrophy, or the loss of specialized glandular cell types such as parietal and chief cells, appears to be critical for cancer progression [15], and it is likely that the fundus is involved either before or after the corpus is already engaged in cancer.

A relatively high positivity for *H. pylori* was found at the tumor-site despite prior statements that *H. pylori* cannot develop in atrophied mucosa and that the intestinal metaplasia is an unfavorable milieu [16]. Tang *et al.*, however, suggested that *H. pylori* might survive in the gastric epithelium in the presence of a carcinogenic lesion [17]. It has also been speculated that certain virulence factors can play important roles in the persistence of *H. pylori* in the gastric mucosa [18].

In the present study, we observed that *H. pylori* demonstrates similar colonization of the anatomic regions of the stomach and tumor site. *H. pylori* positivity in early and advanced gastric cancer groups was also found to be very similar; Tang *et al*., however, observed the inverse results [17]. We believe that the difference observed between these two studies is due to the number of biopsies studied in each patient; we demonstrated that systematic sampling modifies the association of *H. pylori* with illness.

A higher prevalence of *H. pylori cagA* gene-positivity was found in the corpus and fundus of patients with dyspepsia. Previously, Semino-Mora *et al*. showed that this pattern is not present in patients with gastric cancer [19], although we used a different *cagA* amplification fragment in the present work. Gastric neoplastic transformation is induced and sustained, at least in part, by *in vivo* intracellular *cagA* expression; this effect could play a role not only during early gastric carcinogenesis but, based on our data, could also occur later in this process [19]. We observed that 15% of the strains possessed a mixture of *cagA* 3′ repeat region sizes, suggesting the presence of co-infection with multiple *H. pylori* strains, mainly in the non-cancer group. Kidd *et al.* obtained different results; while these authors studied a higher number of patients [20], we studied a higher number of gastric biopsies of which included several specific sites within the stomach.

We previously observed the presence of genotypic diversity in the peptic ulcer corpus [8]. This diversity has been proposed to reflect the synergistic actions that are associated with this process [21]. The predominant *vacA* genotype signal sequence detected in both study groups was s1b, in agreement with our previous report [22].

The important findings of our study are that there is a topographic distribution of the *vacA* signal-sequence, the s1b genotype is commonly associated with the non-cancer group and the s1b/s2 combination or s2 genotypes are found solely in the gastric cancer group. No previous studies on individuals with gastric cancer have considered the four tumor zones evaluated in the present work. The s1c genotype was found to be the most prevalent genotype in Asia; however, its association with gastric cancer is diminishing [22], [23]. The diversity of the *vacA* genotype of *H. pylori* has been explained by a co-evolution model [21].

Regarding *vacA* mid-region alleles, we found m1 and m2 genotypes, in both studied groups, with m1 genotype being the most frequent in the predetermined regions of the evaluated stomach [8].

In both studied groups, we found *H. pylori* strains that were untypeable for the signal-sequence and mid-region alleles due to significant variation in the *vacA* gene [8], [24]. We consistently found that the *cagA*- and *vacA*-allele diversity was greater in the tumor, suggesting that *H. pylori* can remain in the tumor without environmental stress.

The genotypic diversity observed across systematic whole organs and tumors in the present work was remarkable. The principal practical implication of this finding is the need to search for a similar exhaustive sampling scheme in order to associate a pathogenicity factor with disease. We found that the association of a single isolate—derived from a single biopsy—with disease is not sufficient due to the multistrain nature of *H. pylori* infection demonstrated in the present work.

The limitations of this study are related to its observational nature and its cross-sectional design. We cannot exclude the possibility that there is a confounding variable associated both with the presence of the *Helicobacter pylori* infection and the cancer or dyspeptic status. In addition, the lack of a longitudinal follow-up prevents us from confirming the sequential appearance of the events, like cancer development and the initial infection with the strain(s) of *Helicobacter pylori* that were eventually detected.

Conflict of interest statement

The authors declare no conflicts of interest.

## Supporting Information

Figure S1 Topographical signal sequence and middle region allele distribution of the vacA gene in patients with and without gastric cancer.
(3.81 MB TIF)
