# Supplementary figures and images for: High Diversity of vacA and cagA Helicobacter pylori Genotypes in Patients with and without Gastric Cancer
Source: PLoS One. 2008 Dec 3;3(12):e3849. doi: 10.1371/journal.pone.0003849 (PMC2585809; doi:10.1371/journal.pone.0003849)

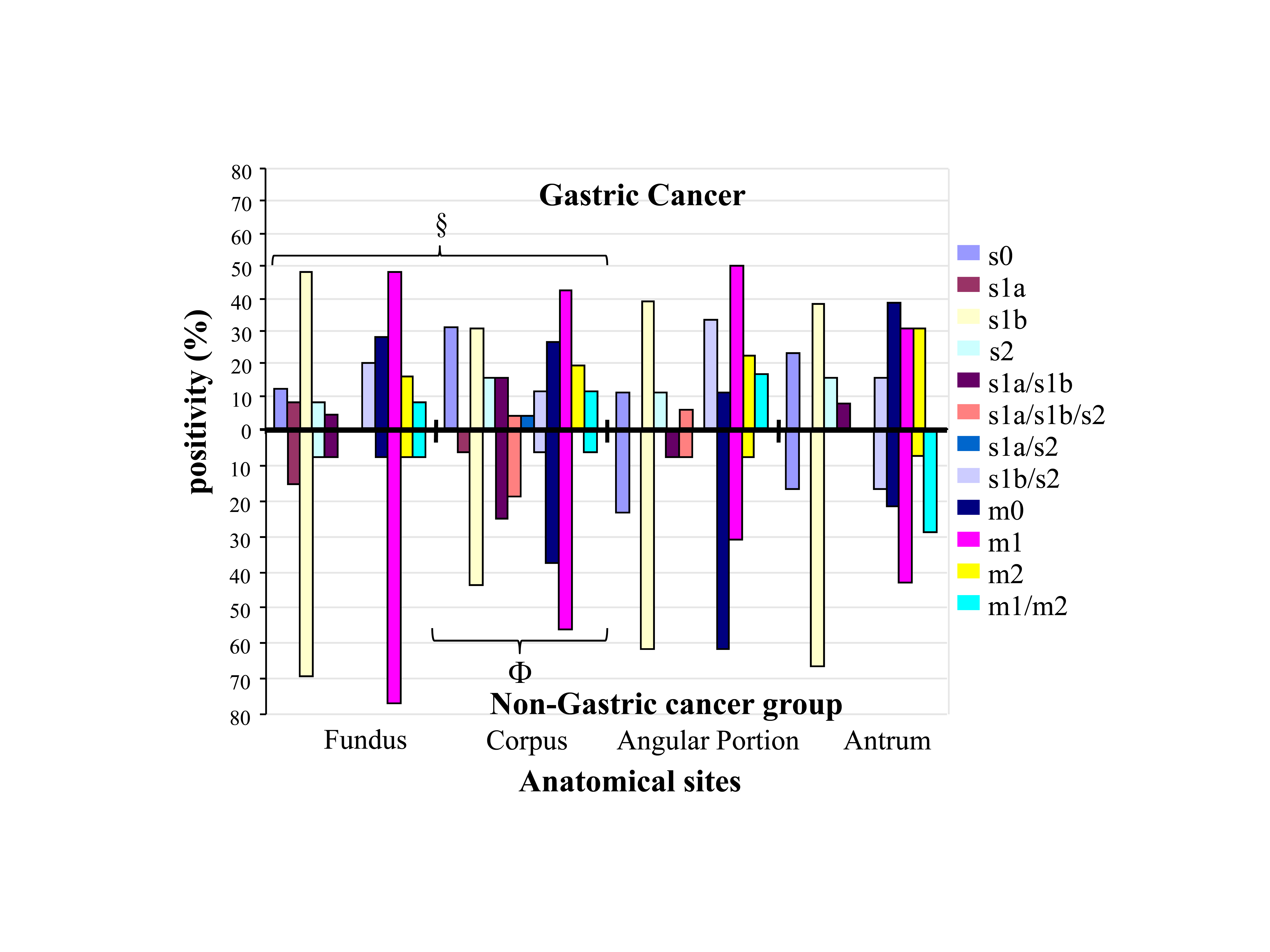

Supplement: Figure S1 — Topographical signal sequence and middle region allele distribution of the vacA gene in patients with and without gastric cancer. (3.81 MB TIF) [file pone.0003849.s001.tif]
